# Apnoea as a novel method to improve exercise performance: A current state of the literature

**DOI:** 10.1113/EP091905

**Published:** 2024-07-19

**Authors:** Janne Bouten, Louise Declercq, Jan Boone, Franck Brocherie, Jan G. Bourgois

**Affiliations:** ^1^ Department of Movement and Sports Sciences Ghent University Ghent Belgium; ^2^ Laboratory of Sport, Expertise and Performance (EA 7370) French Institute of Sport (INSEP) Paris France; ^3^ Centre of Sports Medicine Ghent University Hospital Ghent Belgium

**Keywords:** breath‐hold, erythropoietin, exercise performance, haemoglobin, spleen contraction, voluntary hypoventilation

## Abstract

Acute breath‐holding (apnoea) induces a spleen contraction leading to a transient increase in haemoglobin concentration. Additionally, the apnoea‐induced hypoxia has been shown to lead to an increase in erythropoietin concentration up to 5 h after acute breath‐holding, suggesting long‐term haemoglobin enhancement. Given its potential to improve haemoglobin content, an important determinant for oxygen transport, apnoea has been suggested as a novel training method to improve aerobic performance. This review aims to provide an update on the current state of the literature on this topic. Although the apnoea‐induced spleen contraction appears to be effective in improving oxygen uptake kinetics, this does not seem to transfer into immediately improved aerobic performance when apnoea is integrated into a warm‐up. Furthermore, only long and intense apnoea protocols in individuals who are experienced in breath‐holding show increased erythropoietin and reticulocytes. So far, studies on inexperienced individuals have failed to induce acute changes in erythropoietin concentration following apnoea. As such, apnoea training protocols fail to demonstrate longitudinal changes in haemoglobin mass and aerobic performance. The low hypoxic dose, as evidenced by minor oxygen desaturation, is likely insufficient to elicit a strong erythropoietic response. Apnoea therefore does not seem to be useful for improving aerobic performance. However, variations in apnoea, such as hypoventilation training at low lung volume and repeated‐sprint training in hypoxia through short end‐expiratory breath‐holds, have been shown to induce metabolic adaptations and improve several physical qualities. This shows promise for application of dynamic apnoea in order to improve exercise performance.

## Abstract (highlights)

- **What is the topic of this review?**
  Apnoea is considered as an innovative method to improve performance. This review discusses the effectiveness of apnoea (training) on performance.
- **What advances does it highlight?**
  Although the apnoea‐induced spleen contraction and the increase in EPO observed in freedivers seem promising to improve haematological variables both acutely and on the long term, they do not improve exercise performance in an athletic population. However, performing repeated sprints on end‐expiratory breath‐holds seems promising to improve repeated‐sprint capacity.

## 1 INTRODUCTION

Hypoxic training is a widely used method among athletes to improve sports performance (Girard et al., 2020; Millet et al., 2010) and is, therefore, a popular strategy in the lead‐up to the summer and winter Olympics. A wide range of modalities are used varying from natural and simulated hypobaric and normobaric hypoxia to chronic and intermittent exposure (Treff et al., 2022). In this context, apnoea training could fit within the ‘living low–training high’ hypoxic training methods. Since no equipment is needed, apnoea training can be easily applied during travelling for training camps and competitions.

To protect itself from the detrimental effects of apnoea‐induced hypoxia, the human body elicits a complex interplay of fascinating physiological responses. First, a sympathetically mediated spleen contraction leads to a transient increase in haemoglobin concentration ([Hb]) (Bakovic et al., 2005), the main determinant for oxygen transport and storage in the blood. Performing breath‐holds shortly prior to competition may open opportunities to improve immediate sports performance through initiation of a spleen contraction. Second, apnoea‐induced hypoxia triggers a transient erythropoietin (EPO) response (de Bruijn et al., 2008; Elia, Barlow et al., 2019) through decreased regional oxygen content in the kidney tissue (Wenger & Hoogewijs, 2010), which could improve Hb content on the long‐term and therefore aerobic performance. Given the potential of these responses to increase [EPO] (de Bruijn et al., 2008) and Hb content (Bakovic et al., 2005; Richardson et al., 2005), Lemaître et al. (2010) hypothesized that apnoea could be used as a novel training method to improve endurance performance. More than a decade later, this review aims to provide an update on the effectiveness of apnoea training on performance based on the current scientific literature, and expand on the potential for restricted breathing during exercise.

## 2 EFFECT OF APNOEA‐INDUCED SPLEEN CONTRACTION ON AEROBIC PERFORMANCE

**FIGURE 1:**
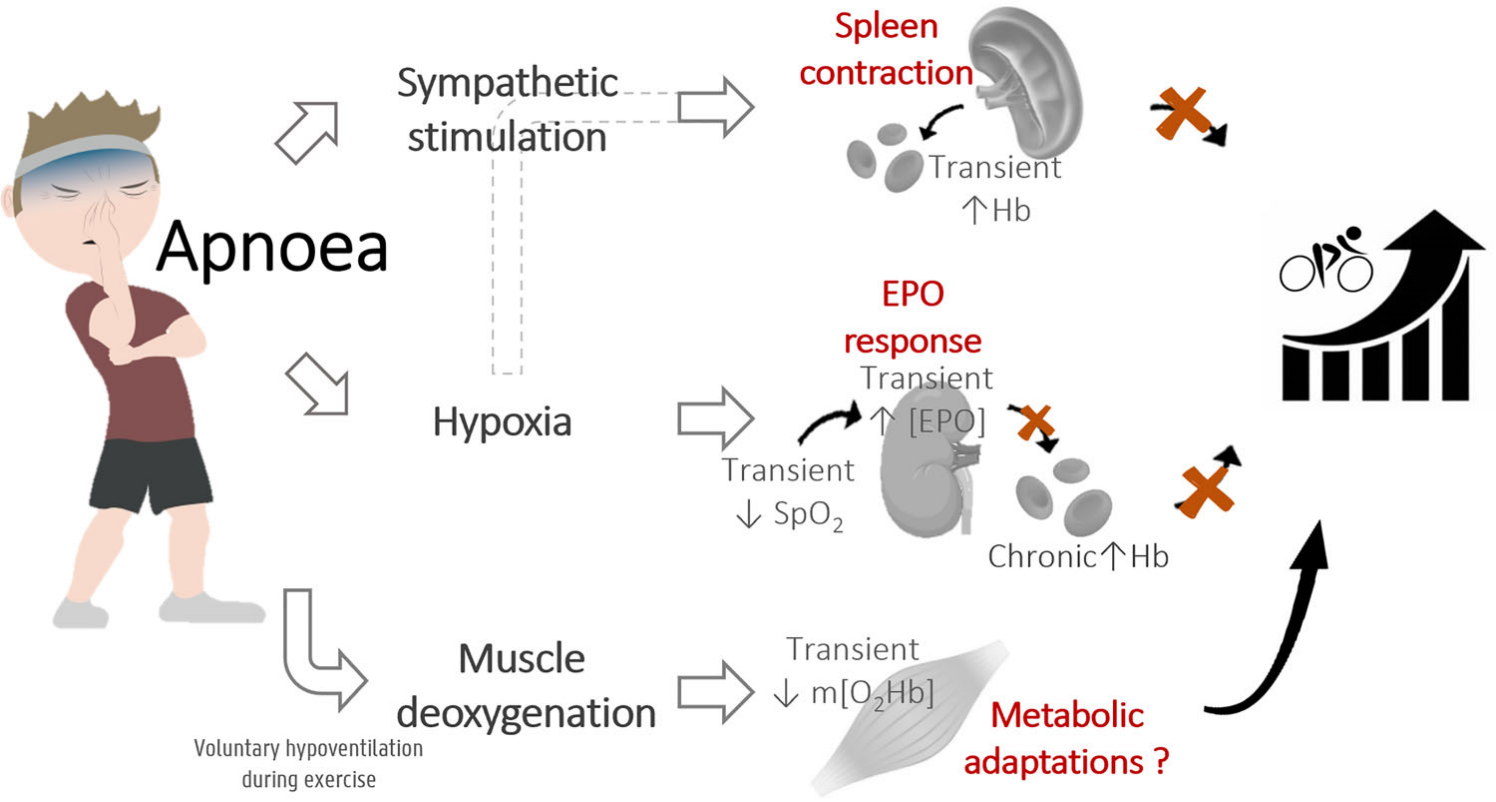
Mechanisms to improve performance through apnoea. Apnoea evokes a spleen contraction (further supported by hypoxia) leading to a transient increase in haemoglobin concentration ([Hb]) which does not translate into improved performance. Decreased peripheral oxygen saturation ($S_{pO_{2}}$) only leads to transient increases in erythropoietin (EPO) in trained freedivers and not individuals without apnoea experience. Apnoea training therefore does not lead to improved Hb and performance. Strong muscle deoxygenation (m[O_2_Hb]) during training with restricted breathing at a lower intensity or during sprint leads to improved performance, most likely induced by metabolic adaptations.

A first opportunity to improve sports performance through breath‐holding is by exploiting the spleen contraction and concomitant transient increase in circulating Hb (Figure 1, upper part). At rest, the spleen rhythmically dilates and contracts. During apnoea, however, a fast and immediate active spleen contraction has been demonstrated in both apnoea‐trained and untrained participants (Baković et al., 2003). This results from a centrally mediated feed‐forward mechanism and is supported by the changes in spleen volume in response to sympathetic nerve stimulation of the spleen (Ayers et al., 1972). Indeed, around 98% of the splenic nerve fibres are sympathetic, while adrenoceptors are located in both the parenchyma and splenic capsule (Udroiu, 2017). As catecholamines are correlated with the decrease in spleen volume in response to apnoea (Shephard, 2016), and blocking α‐adrenoceptors reduces the effect of both sympathetic nerve stimulation and injection of noradrenaline in humans (Ayers et al., 1972), spleen contraction is expected to be sympathetically mediated through stimulation of α‐adrenoceptors. This response might possibly be supported by a passive collapse due to reduced splenic blood flow as described by Allsop et al. (1992). Indeed, an immediate strong spleen contraction followed by a steady spleen volume reduction until the end of apnoea has been previously observed in trained freedivers (Baković et al., 2003), with a further smaller reduction in spleen volume throughout a series of breath‐holds in both apnoea trained and untrained participants (Bouten et al., 2019; Schagatay et al., 2020). However, this is not consistent across studies (Baković et al., 2003).

Since the spleen also contracts in response to intense exercise, it is believed to be an important contributor to exercise performance in horses and dogs (Shephard, 2016). In humans, it has been shown that acute apnoea increases [Hb] by 2%–5% on average and up to 13 g L^−1^ individually (Bouten et al., 2019). Considering that each gram of Hb binds 1.34 mL of oxygen, while the amount of oxygen dissolved in the blood is trivial, it is clear that Hb is the main determinant for oxygen transport and storage in the blood. Hence, a strong correlation (*R* = 0.91) between haemoglobin mass (Hb_mass_) and maximal oxygen uptake (${\dot{V}}_{O_{2}\max}$) has been observed with each increment of 1 gram in Hb_mass_ corresponding to an improvement in ${\dot{V}}_{O_{2}\max}$ by approximately 4 mL min^−1^ (Schmidt & Prommer, 2010). Assuming a blood volume of 5 L, an absolute increase in ${\dot{V}}_{O_{2}\max}$ of up to 260 mL min^−1^ immediately after apnoea due to splenic contraction might be anticipated. As such, the athletes may start the competition with improved oxygen‐carrying capacity which might enhance oxygen uptake (${\dot{V}}_{O_{2}}$) kinetics and aerobic performance, potentially resembling the performance‐enhancing effects observed with blood transfusion (Bejder et al., 2019). However, it is still under debate to what extent the increase in [Hb] is related to splenic contraction or to a decrease in plasma volume. Furthermore, measuring splenic volume via ultrasound is also methodologically challenging. This leads to a large range of splenic volume reductions observed ranging from 30 to 100 mL (Baković et al., 2003; Bouten et al., 2019; Holmström et al., 2021; Lindblom et al., 2024; Lodin‐Sundström & Schagatay, 2010; Robertson et al., 2020).

Sperlich et al. (2015) were the first to test whether acute apnoea improves performance. Despite a significant, yet small, reduction in spleen volume after a series of four breath holds, they failed to observe a positive effect on a 4 km cycling time trial in trained cyclists (${\dot{V}}_{O_{2}\mathrm{peak}}$ = 56.6 ± 6.6 mL min^−1^ kg^−1^). Contrarily, the participants needed an extra 16 s to complete the 4 km cycling time trial following the apnoea intervention. Interestingly, the participants already lost 7 s in the first kilometre. The short time interval (30–45 s) between the apnoea intervention and the cycling time trial was probably not sufficient for the participants to recover physically and mentally. Robertson et al. (2020) used a longer rest interval (2 min) but also did not observe differences for a 400 m swimming performance in trained to highly trained swimmers (regional/national level) with and without a prior series of three apnoea interspersed by 2 min rest intervals. However, Wendi et al. (2024) found a 6–8% higher peak power output and ${\dot{V}}_{O_{2}\mathrm{peak}}$ during an incremental exercise test in highly trained rugby players after a warm‐up with end‐expiratory dynamic apnoeas compared to normal warm‐up. However, the 7% difference in peak heart rate raises questions about the quality of the incremental exercise tests to determine peak power and ${\dot{V}}_{O_{2}\mathrm{peak}}$. Additionally, the increase in ${\dot{V}}_{O_{2}\mathrm{peak}}$ was not accompanied by a significant increase in [Hb] or red blood cell count, when comparing the dynamic apnoea warm‐up protocol with the normal breathing warm‐up (Wendi et al., 2024). Furthermore, neither of the other studies observed a transient apnoea‐induced increase in [Hb] (Robertson et al., 2020; Sperlich et al., 2015), which might be related to the rather low reductions in splenic volume (Sperlich et al., 2015). This raises the question whether the appropriate apnoea protocols were applied but also adds further doubts whether splenic contraction is a valid contributor to improve performance following acute apnoea.

To answer these questions and counter potential limitations, Bouten, Colosio et al. (2020) examined the efficiency of different apnoea modalities on the Hb response and perceived readiness for subsequent strenuous exercise in trained participants without experience in apnoea. Static and maximal apnoeas turned out to evoke the strongest Hb response while no significant difference was observed for the number of apnoeas performed. Moreover, participants indicated higher scores of readiness to perform in exercise after only one compared to a series of five apnoeas. The authors therefore suggested that one maximal static breath‐hold performed shortly before the start of the exercise appeared to be the most promising when both physiological and psychological factors are considered (Bouten, Colosio et al., 2020). One single apnoea is also more easily applicable in a real‐life sports competition, explaining why this protocol was chosen to test the effect of apnoea‐induced spleen contraction on performance. This one maximal apnoea protocol tended to improve ${\dot{V}}_{O_{2}}$ kinetics compared to a control condition (Bouten, Colosio et al., 2020). However, in another study, the amplitude of the spleen contraction during exercise did not correlate with kinetics (Zubac et al., 2022). Despite potential faster ${\dot{V}}_{O_{2}}$ kinetics, there was no difference in 3 km time trial performance nor power output between the apnoea, control and placebo conditions when the apnoea protocol was integrated in a warm‐up (Bouten, Colosio et al., 2020). Bearing in mind that the target population for this apnoea method is highly trained to elite athletes who are accustomed to exercise but not to apnoea, it is likely that the spleen will respond more strongly to exercise, during both the warm‐up and the actual time trial, than to apnoea (Holmström et al., 2021). Lindblom et al. (2024) recently confirmed this hypothesis in trained and highly trained cross‐country skiers (${\dot{V}}_{O_{2}\max}$ > 60 mL min^−1^ kg^−1^) showing a progressively stronger spleen contraction and [Hb] increase for higher exercise intensities. Furthermore, the higher intensities elicited a stronger spleen contraction than an apnoea protocol consisting of three maximal static apnoeas (Lindblom et al., 2024). A well‐designed warm‐up therefore likely overrules the potential beneficial effects (e.g., transient [Hb] increase and improved ${\dot{V}}_{O_{2}}$ kinetics) of the apnoea‐induced spleen contraction. This assumption is supported by the small improvement in performance observed with the combination of apnoea and warm‐up, but not with apnoea alone in 400 m swimming performance in trained to highly trained swimmers (Robertson et al., 2020), although this could also indicate that the amplitude of the spleen response to apnoea in humans might just be too small to affect exercise performance. The only study so far who observed performance enhancement after acute apnoea (Bourdas & Geladas, 2021) did not use an effective warm‐up. Participants improved time to exhaustion while cycling at 150% of peak power output from 44.8 to 49.2 s (+11%) after a series of five apnoeas compared to a control condition. Unfortunately, the time to exhaustion type of test is less reliable than fixed duration or time trial tests (Jeukendrup et al., 1996). Furthermore, all participants performed the control condition first and the apnoea condition the week after, without mention of any familiarization session in the study design. Therefore, the 5 s performance improvement might just stem from habituation to the protocol, especially in a non‐cycling population that was not well trained (${\dot{V}}_{O_{2}\max}$ of 42.2 mL min^−1^ kg^−1^) (Bourdas & Geladas, 2021).

Cross‐sectional data on direct comparison of spleen volume between trained breath‐hold divers and untrained individuals usually do not report differences (Baković et al., 2003; Elia et al., 2021b, Elia, Wilson et al., 2019; Prommer et al., 2007). However, the Bajau, a population who uses freediving daily for economic purposes, have larger spleens than their non‐diving counterparts (Ilardo et al., 2018). This was not observed for Japanese Ama, but their control group was considerably taller and heavier, which might influence the results (Hurford et al., 1990). Although differences have been linked to genetics (Ilardo et al., 2018), larger spleen volumes in elite biathletes (Holmström et al., 2021) and climbers (Schagatay et al., 2020) might indicate an effect from frequent exposure to hypoxia and high‐intensity exercise, a combination that is known to evoke a stronger spleen contraction than hypoxia alone (Lodin‐Sundström et al., 2021; Schagatay et al., 2020). However, genetic predispositions for their respective activities cannot be excluded in these populations. Increased baseline spleen volumes following 8 weeks of static (Bouten et al., 2019) and dynamic (Bouten et al., 2019; Yang et al., 2022) apnoea training further suggest a potential training or exposure effect. However, this was not confirmed in another study including 6 weeks of dynamic apnoea (Elia et al., 2021a). Furthermore, although not always consistent (Elia et al., 2021b), both trained freedivers (Baković et al., 2003; Richardson et al., 2005) and elite biathletes (Holmström et al., 2021) have shown stronger spleen contractions in response to apnoea than recreationally active individuals. It could therefore be argued that the acute apnoea‐induced increase in [Hb] and therefore the potential of the apnoea‐induced spleen contraction to enhance aerobic performance might be improved after apnoea training. Indeed, although apnoea alone is a sufficient stimulus for spleen contraction, hypoxia itself is also known to induce a spleen contraction (Lodin‐Sundström & Schagatay, 2010; Persson et al., 2023). A stronger hypoxic stimulus during longer apnoea might therefore help develop spleen contraction towards the end of apnoea. A recent study measuring haematocrit and red blood cell concentration using capillary blood samples, reported an improved haematological response to a series of five maximal apnoeas after 2 weeks of static apnoea training in recreationally active participants (Bourdas & Geladas, 2023). However, studies using ultrasonography and venous blood samples have up to now failed to observe a stronger spleen contraction and increased [Hb] response after 2 (Engan et al., 2013) and 8 weeks (Bouten et al., 2019) of static apnoea training. Furthermore, cycling time to exhaustion at 150% of peak power output after a series of five apnoeas did not improve following 2 weeks of apnoea training (Bourdas & Geladas, 2021).

Based on the current literature (Bouten, Colosio et al., 2020; Robertson et al., 2020; Sperlich et al., 2015), growing evidence shows that apnoea does not provide any additional benefit to, nor is a valid alternative for, a warm‐up. From a physiological perspective, the peripheral vasoconstriction and decrease in muscle metabolism that occur during apnoea (Eichhorn et al., 2015) are the opposite of what is expected with a standard active warm‐up. Additionally, muscle and brain tissue deoxygenate during apnoea (Bouten, Bourgois et al., 2020; Eichhorn et al., 2015) while partial pressure of CO_2_ builds up (hypercapnia) and pH decreases (Willie et al., 2015). Furthermore, athletes experiencing strenuous involuntary breathing movements might also induce fatigue in their respiratory muscles (Batinic et al., 2016). It is currently unknown how much time is needed for these possible negative effects to fade. Given the physical discomfort that comes with prolonged apnoea, decreased perceived readiness to perform intense exercise can also not be ignored.

## 3 EFFECT OF APNOEA‐INDUCED EPO RESPONSE ON AEROBIC PERFORMANCE

Based on the transient increases in [EPO] following breath‐holds in trained freedivers (de Bruijn et al., 2008; Elia, Barlow et al., 2019; Elia et al., 2021a; Kjeld et al., 2015), increasing baseline Hb with apnoea training might be an additional opportunity to improve aerobic performance (Figure 1, middle part). During apnoea, the continuous flow of oxygen to the lungs gets interrupted and the body has to rely on its intrinsic oxygen stores, which are steadily depleted. Indeed, trained breath‐hold divers regularly reach peripheral oxygen saturation ($S_{pO_{2}}$) values below 80% during breath‐holding, while values below 60% have also been reported (de Bruijn et al., 2008; Kjeld et al., 2021). Hypoxia‐inducible factors (HIFs) are known to coordinate the transcriptional responses during hypoxia in the cell at different levels (Haase, 2013). HIFs are heterodimer transcription factors consisting of a HIFα and HIFβ subunit. HIFβ is continuously expressed in the cells, while HIFα levels are highly oxygen‐dependent. Under normal well‐oxygenated conditions, HIFα quickly gets degraded. However, under hypoxic conditions, HIFα degradation is inhibited. HIFα levels then accumulate which allows for heterodimerization with the HIFβ subunit to form a fully functioning HIF transcription factor complex. HIF transcriptional activity then leads to the expression of hypoxia response elements (HRE) in the promotor regions of the genes. HIFα has two isoforms, HIF1α and HIF2α, with HIF2α being the main factor involved in the regulation of [EPO] (Haase, 2013). Serum EPO levels therefore increase in a dose‐dependent manner in response to reductions in $S_{pO_{2}}$ (Chapman et al., 2014). Unfortunately, the impact of the apnoea‐induced hypoxia on the stabilization of HIF2α, formation of HIF2 heterodimers and upregulation of transcriptional activation has never been evaluated, but acute increases in [EPO] following apnoea have been observed (de Bruijn et al., 2008; Kjeld et al., 2015).

Longitudinally, there are some indications that aerobic performance could be improved in non‐apnoea‐trained individuals following breath‐hold training. One study reported higher ${\dot{V}}_{O_{2}\mathrm{peak}}$ during an incremental cycling test after 3 months of dynamic apnoeas with 30 s breath‐holds interspersed with 30 s of normal breathing while cycling at 30% of ${\dot{V}}_{O_{2}\mathrm{peak}}$ (Lemaitre et al., 2009). However, only four participants were included and a training effect (due to 3 months of low‐intensity cycling sessions in participants previously not familiar with cycling) might have biased the results (Lemaitre et al., 2009). Indeed, a similar protocol did not lead to changes in ${\dot{V}}_{O_{2}\mathrm{peak}}$ in triathletes (Joulia et al., 2003). Concerning haematological parameters, increased reticulocyte counts have been observed after 2 weeks of static (Engan et al., 2013) and 6 weeks of dynamic apnoea training (Elia et al., 2021a). The effect on [Hb] currently shows mixed results with some studies observing no changes after 2 weeks of static (Bourdas & Geladas, 2023; Engan et al., 2013) and 6 (Elia et al., 2021a) or 8 weeks of dynamic apnoea training (Yang et al., 2022), while 8 weeks of static apnoea training led to a small yet significant increase in [Hb] (Bouten et al., 2019). Studies so far have mostly focused on [Hb] (Bouten et al., 2019; Elia et al., 2021a; Engan et al., 2013; Yang et al., 2022). However, [Hb] is highly affected by plasma volume, leaving conclusions highly susceptible to methodological issues.

Only two studies investigated the change in Hb_mass_, either following a 6‐week apnoea training intervention compared in recreationally active individuals without apnoea experience (Bouten et al., 2022) or throughout an 8‐month follow‐up in recreational freedivers (Astolfi et al., 2022). Bouten et al. (2022) combined haematological and performance parameters in order to highlight the underlying physiological mechanisms involved in potential aerobic performance improvements. The results were straightforward with no change observed in Hb_mass_, ${\dot{V}}_{O_{2}\mathrm{peak}}$ nor 3 km time trial performance, in neither the apnoea training group nor the control group (Bouten et al., 2022). Regarding the underlying physiological parameters, no effects on reticulocytes and more importantly [EPO] could be observed, both acutely and for baseline values (Bouten et al., 2022). Similarly, Hb_mass_ does not seem to be affected by apnoea training in recreational breath‐hold divers (Astolfi et al., 2022).

Elia, Barlow et al. (2019) found acute increases in [EPO] upon both dynamic and to a lesser extent static apnoea in trained freedivers but not in inexperienced participants. The decreases in $S_{pO_{2}}$ (82–96%) during apnoea in these inexperienced participants (Bouten et al., 2022; Elia, Barlow et al., 2019) seem less pronounced than in trained freedivers (73–76%) (De Bruijn et al., 2008; Elia et al., 2021b) indicating the need for stronger oxygen desaturation. Furthermore, the time spent in hypoxia was also significantly longer in freedivers. For instance, freedivers spent on average 12 min 25 s at; $S_{pO_{2}}$ below 85% (De Bruijn et al., 2008), which is similar or even longer than the total apnoea time performed by inexperienced participants in either one (Bouten et al., 2022) or two series of five apnoeas (Elia, Barlow et al., 2019). The hypoxic dose, that is, the combination of time spent in hypoxia and magnitude of desaturation (Millet et al., 2016), is most likely insufficient in non‐apnoea‐trained individuals, even after 6 weeks of static apnoea training (Bouten et al., 2022). Moreover, data from Elia et al. (2021a) undermine the idea that this could be improved by training. They observed acute increases in [EPO] following a dynamic apnoea training session in untrained participants, but this acute EPO increase was attenuated following 3 and 6 weeks of apnoea training despite improved maximal apnoea times. This might be related to the hypothesis that hypercapnia suppresses HIF2α accumulation and HIF‐dependent transcriptional activity in the presence of hypoxia (Selfridge et al., 2016). Furthermore, although some cross‐sectional studies have indicated larger [Hb] values in trained freedivers or diving populations (Elia, Wilson et al., 2019 Kang et al., 1963; Richardson et al., 2005), most do not show differences in baseline [Hb] (Bakovic et al., 2005; Baković et al., 2003; Kjeld et al., 2018) or Hb_mass_ (Prommer et al., 2007). Additionally, similar seasonal changes in haematological parameters (e.g., [Hb], reticulocytes, Hb_mass_) between recreational freedivers and controls throughout an 8‐month longitudinal follow‐up have been reported (Astolfi et al., 2022). This further supports the idea that the hypoxic dose induced by apnoea might be insufficient to induce erythropoiesis, even in trained freedivers.

Aside from the potential benefits of apnoea training for aerobic performance, one must also be aware of risks and possible unexpected training effects. Although debated (Dujic et al., 2008), Roecker et al. (2014) warned of a possible reduced aerobic capacity due to a lowered ventilatory response to CO_2_ (Costalat et al., 2014; Roecker et al., 2014). They showed that this leads to depressed ventilation at all exercise intensities and a metabolic shift toward greater anaerobic and depressed aerobic energy supply (Roecker et al., 2014). Furthermore, although the diving response is effective at maintaining brain oxygenation and protecting the body against the negative effects of apnoea‐induced hypoxia (Bouten, Bourgois et al., 2020; Eichhorn et al., 2015), hypoxic black‐out cannot be excluded (Bouten, Bourgois et al., 2020 Mulder et al., 2023). Additionally, the simultaneous stimulation of sympathetic and parasympathetic activity in face‐immersed and/or dynamic apnoeas, the so‐called autonomous conflict (Shattock & Tipton, 2012) is known to evoke arrhythmias. However, this does not seem to translate to adverse cardiac events in healthy populations frequently performing apnoeas (Doerner et al., 2018; Tanaka et al., 2016; Zelenkova & Chomahidze, 2016). As such, experienced supervision is recommended.

## 4 RESTRICTED BREATHING DURING EXERCISE

The present review clearly indicates that, from a haematological point of view, apnoea (training) does not appear to be useful to improve aerobic performance. However, applying breath‐holding differently, that is, integrated in regular training sessions, may reveal some benefits for improving performance indicators (Figure 1, lower part). First, restricted breathing frequency, also called voluntary hypoventilation, during swimming, either at high (Karaula et al., 2016; Lavin et al., 2015) or at low (Trincat et al., 2017; Woorons et al., 2016) end‐expiratory lung volume, has been shown to improve 100 m to 400 m swimming performance (Karaula et al., 2016; Lavin et al., 2015; Woorons et al., 2016) but not 50 m performance (Lemaitre et al., 2009) nor ${\dot{V}}_{O_{2}\mathrm{peak}}$ (Lavin et al., 2015; Woorons et al., 2008, 2016). This method is effective in eliciting both hypoxic and hypercapnic stress although it is difficult to discern their respective effects (Woorons et al., 2010). Further, six sessions of voluntary hypoventilation while cycling at 150% of mean power output of a 3‐min ‘all‐out’ test improved performance during a Yo‐Yo intermittent recovery test level 1 in trained team‐sport athletes (Woorons et al., 2020). Second, implementation of voluntary hypoventilation at low lung volume during repeated sprints seems even more promising. Original studies show that as little as six sessions of repeated‐sprint training with end‐expiratory breath‐holding (the so‐called RSH‐VHL) have proven beneficial in improving repeated‐sprint ability in running, cycling, swimming and team‐sport athletes (Brocherie et al., 2023; Fornasier‐Santos et al., 2018; Lapointe et al., 2020; Trincat et al., 2017; Woorons et al., 2019, 2020).

Although the exact mechanisms still remain largely unknown, metabolic changes most likely cause the above‐mentioned improvements as a consequence of exaggerated muscle deoxygenation during breath‐holds (Woorons et al., 2017) due to limited oxygen storage in near‐empty lungs and possible peripheral vasoconstriction. Improved inspiratory and/or expiratory muscle strength (Karaula et al., 2016; Lavin et al., 2015), increased muscle buffer capacity (Woorons et al., 2008), anaerobic glycolysis (Woorons et al., 2016) and oxygen utilization in fast‐twitch fibres have also been suggested. The latter seems similar to adaptations in traditional repeated‐sprint training in hypoxia (Brocherie et al., 2017) but has thus far not been confirmed in research (Woorons et al., 2019) in which recovery occurs during normoxia while hypercapnic stress is potentially added during sprints. This was also suggested in a recent meta‐analysis which claims to support changes in anaerobic (lactate) but not aerobic (${\dot{V}}_{O_{2}\mathrm{peak}}$) markers (De Asís‐Fernández et al., 2022). Unfortunately, only lactate and ${\dot{V}}_{O_{2}\mathrm{peak}}$ were addressed in this meta‐analysis. Furthermore, effects of maximal static and short high‐intensity dynamic apnoeas were pooled for ${\dot{V}}_{O_{2}\mathrm{peak}}$ while both methods target very different physiological adaptations (haematological vs. peripheral). Voluntary hypoventilation at low lung volume at lower intensity and during sprinting was also pooled for the effect on lactate. In our view, conclusions from this meta‐analysis therefore need to be interpreted with caution. Lastly, end‐expiratory breath‐holds present a unique combination of apnoea, high‐intensity exercise and hypoxia, three factors known to independently and simultaneously evoke spleen contractions (Lindblom et al., 2024; Lodin‐Sundström & Schagatay, 2010; Lodin‐Sundström et al., 2021; Persson et al., 2023). A beneficial training effect of these repeated sprints at end‐expiratory breath‐holds on the function of the spleen affecting exercise performance might be hypothesized. However, this has never been addressed scientifically before.

## 5 CONCLUSIONS

Static breath‐holds shortly prior to competition may be beneficial for immediate sports performance when a warm‐up is not possible, for instance when the competition format requests long waiting periods (e.g., track and field events, team‐sport substitute players). It has been suggested that apnoea could mimic a high‐intensity warm‐up by affecting the acid–base response through acceleration of oxygen consumption (Bourdas & Geladas, 2021). However, scientific evidence is scarce and the relevance of splenic contraction during exercise is still debated. In contrast, when a warm‐up is possible, static apnoea seems redundant since a warm‐up has already beneficial effects (Bouten, Colosio et al., 2020; Robertson et al., 2020).

Breath‐hold training does not seem to improve haematological variables nor aerobic performance in apnoea novices and remains questionable in apnoea‐trained individuals. However, restricted breathing during exercise, and especially end‐expiratory breath holds, could improve anaerobic performance, particularly in activities that require repeated sprints (Fornasier‐Santos et al., 2018; Lapointe et al., 2020; Trincat et al., 2017; Woorons et al., 2019, 2020). A 3‐ to 4‐week repeated‐sprint training period with end‐expiratory breath‐holds is typically recommended, but even a few sessions have been demonstrated to be effective. This type of training could provide a practical and low‐cost alternative training method for elite and world‐class athletes preparing for major events such as the Olympic Games. A special focus should be placed on further unravelling the underlying mechanisms of improved anaerobic performance and comparing the training outcomes to traditional hypoxic training.

## AUTHOR CONTRIBUTIONS

Janne Bouten, Franck Brocherie and Jan G. Bourgois conceptualized the paper. Janne Bouten wrote the original draft of the paper. Louise Declercq, Jan Boone, Franck Brocherie and Jan G. Bourgois critically reviewed and edited the paper, involving reviewing material and adding new content throughout. All authors wrote, drafted and critically revised the paper throughout its inception. All authors have read and approved the final version of the manuscript. All authors agree to be accountable for all aspects of the work in ensuring that questions related to the accuracy or integrity of any part of the work are appropriately investigated and resolved. All persons designated as authors qualify for authorship, and all those who qualify for authorship are listed.

## CONFLICT OF INTEREST

None.
